# Consensus Report of the XI Congress of the Spanish Society of Odontology for the Handicapped and Special Patients

**DOI:** 10.4317/medoral.19569

**Published:** 2014-03-08

**Authors:** Guillermo Machuca-Portillo, Carmen Cabrerizo-Merino, Antonio Cutando-Soriano, María J. Giménez-Prats, Farncisco J. Silvestre-Donat, Inmaculada Tomás-Carmona

**Affiliations:** 1 Senior Lecturer of Special Care in Dentistry. Faculty of Odontology. University of Sevilla, Spain; 2 Medical doctor. Stomatologist. USBD Medical Center of Ranero. Associate professor of Docent Unit of Special Patients. University Odontological Clinic. Faculty of Medicine. Murcia, Spain; 3 Senior Lecturer of Special Care in Dentistry. Faculty of Odontology. University of Granada, Spain; 4 Medical Doctor. Medical Director of the Hospital “Nen Deu” Barcelona, Spain; 5 Professor of Special Care in Dentistry. Faculty of Odontology. University of Valencia, Spain; 6 Senior Lecturer in Special Care in Dentistry. Faculty of Medicine and Odontology. University of Santiago de Compostela, Spain

## Abstract

This article summarizes the findings of consensus of the XI congress of the SEOEME. All of these conclusions are referring to the review articles responsible to the general rapporteurs in order to bringing up to date knowledge with regard to the use of implants in patients medically compromised and with special needs and, in the dental management of autism and cerebral palsy, in the dental treatment of patients with genetic and adquired haematological disorders, the dental implications of cardiovascular disease and hospital dentistry.

** Key words:**Autism, cardiovascular diseases, cerebral palsy, dental implants, disabled patients, haematological disorders, hospital dentistry.

## Consensus report of group a: dental implants in patients with disabilities and with systemic diseases

The range of indications of dental implants have been very extended in recent times. One of the main factors responsible for this enlargement in the indications lies in the possibility of placing them in patients with systemic pathology. Very recently, many of these cases were regarded as absolute contraindications. The consensus conclusions about the placement of implants in patients with disabilities and systemic diseases are transcribed.

-Can be placed dental implants in psycho-physical disabled patients? (1)

1) The comparison with studies involving other patient populations without mental or physical impediments did not show statistically significant differences in terms of the failure rate recorded.

2) The most important aspects in assessing the predictable aspects of treatment are alien to the intellectual capacity, such as the quantity and quality of bone, possibilities of maintenance of a proper oral hygiene, or control of parafunctions.

3) It is necessary to evaluate each case individually, following a strict surgical protocol and frequent checkups, as well as informing the patient’s caregivers about the importance of maintaining good oral hygiene and the absence of oral habits.

4) Implant therapy provides an increase in patient self-esteem from an aesthetic point of view as well as in their quality of life, reduced by other diseases.

5) More studies with bigger sample and further follow-ups should be carried out, with detailed information about the systemic condition of each patient, the presence of parafunctions, hygiene and placement of the implants. Although more experience is needed, implant rehabilitation can be now considered a suitable option in people with disabilities.

-Which are the contraindications to put dental implants in medically compromised patients? (2)

1) The evidence level of implant failures in medically compromised patients is limited due to the short number of controlled randomised studies. In geriatric medically compromised patients (70 years and over), with controlled systemic diseases, should not be considered as a risk factor for dental implants failure subjected to prosthetic charge.

2) It does not seem to exist correlation between the lack of osseointegration of dental implants and patients with cardiovascular disease.

3) Head and neck radiotherapy could be responsible in the reduction of the success rate of dental implants when it is administered in doses exceeding 50 Gy.

4) The consumption of tobacco seems to be a factor associated with the increase in the loss of dental implants (failure rate 2.5-2.6), although, it is not a matter without controversy.

5) The risk of an increase in failures in diabetic patients seems to be relatively higher, but the risk of augmenting the failure rate are reduced if there is control of diabetes, antibiotic prophylaxis protocol and aseptic techniques with chlorhexidine.

6) Osteoporosis may not be a contraindication for the placement of dental implants. The treatment with intravenous bisphosphonates present bigger incidence of risk of suffering osteonecrosis. The consumption of oral biphosphonates by patients who suffer from osteoporosis seems to be a partial contraindication for the treatment with dental implants.

## Consensus report of group b: dental management of patients with autism and cerebral palsy

-What aspects are considered in reaching the dental management of patients with Autism Spectrum Disorders (ASD)? (3)

1) Usually, patients diagnosed with autism disorder are significantly less cooperative than those diagnosed with Asperger’s syndrome and Pervasive Development Disorder. When older is a patient, higher his or her the cooperation will be. Patients diagnosed with intellectual disability, cerebral paralysis, self-mutilation, or Pica disorder have a higher probability of poor cooperation as opposed to patients without these associated pathologies.

2) There is no protocol for behavior management applicable to all patients. It is recommend that this information is gathered in a preliminary interview first contact with the parents/guardians of the patient.

3) There is no one system applicable to all patients diagnosed with Autism Spectrum Disorders. The most frequently used are: visual pedagogy, behavioural techniques, behaviour modification techniques, physical focus techniques, and sometime pharmacological behavior management techniques.

4) Patients with ASD do not present any specific dental characteristic in the soft or hard tissues, but… 

a. Some authors even reported a lower prevalence of caries.

b. Bruxism is present in between 20 and 60%.

c. Gingivo-periodontal pathology is more prevalent in these patients (differences are explained by the poorer levels of oral hygiene).

d. The presence of adverse effects on the oral cavity from medicines have also been described, particularly hyposalivation, oral ulcers, delayed scarring or gingival enlargement.

-Which are the features of the dental treatment of patients with cerebral palsy compared with those without it? (4)

1) These patients have a higher rate of caries and bacterial plaque in deciduous and permanent teeth compared to the general population. The factors are related to:

a. Difficulty on controlling oral hygiene.

b. The soft diet they follow.

c. Difficulties in chewing and swallowing.

2) The periodontal status is related to an increased frequency of gingival hypertrophy, as a consecuence from the decreased me-chanical action of crushing food, and with the consumption of antiepileptic drugs.

3) These patients have a greater presence of hypoplasia and injuries to upper front teeth, due to the increase in falls caused by lack of motor control. And a high percentage of bruxism, (40-70% of cases), primarily affecting the teeth of the upper jaw and the lower molars and premolars.

4) The most common malocclusion is Class II with open bite and overjet due to the muscular hyperextension of the head which causes stretching of the oral soft tissues that contributes to mandibular retrognathia and vertical growth, causing molars to over-erupt and favoring low tongue position.

5) The drooling is attributed to poor coordination during the voluntary phase of swallowing.

6) A significant proportion of patients with cerebral palsy have to be treated using techniques of moderate-deep sedation or gen-eral anesthesia.

## Consensus report of group c: dental management of patients with genetic and acquired hematological diseases

-What attitude should be taken with the bleeding disorders in the dental office? (5)

1) In the congenital / hereditary and acquired bleeding disorders, substitution treatment, associated with preventive and local hemostasis strategies, are usually enough to control the complications that may arise.

2) The systematic care for these patients may include:

a. Clinical history.

b. Consultation to the specialist (hematologist / internist) if applicable.

c. Considering the style of treatment (ambulatory, outpatient sedation or under general anesthesia), where doing it and if there is possibility to do it all in one session.

d. Prevent nerve block anesthesia techniques, (in particular the inferior alveolar) if feasible.

e. In odontopediatric treatments.

i. Not overused the instrumental in pulpotomy and pulpectomies, 

ii. Do not invade gums (supragingival pediatric dental crown).

iii. Cleaning carefully if there’s granulation tissue under the root.

f. In relation to aspiration.

i. Support the nozzle on rollers or gauze and never directly on mucous.

ii. Using spit ejector suction instead of surgical suction for being less traumatic.

g. According to the use of the rubber dam:

i. Produces gingival retraction and field isolation, 

ii. Reduces the possibility of injuring lips and mucous membranes.

h. In orthodontic treatments, 

i. Bands, brackets and fixed maintainers, always cemented in a supragingival way, 

i. When there are decays, extractions are the last option, 

j. Application of topical hemostasis ways and local measures.

i. Systemic and topical tranexamic acid, 

ii. Desmopressin, 

iii. Material to fill the socket: Surgicell, cellulose plugs, fibrin sponge, microfibrillar collagen, fibrin , cyanoacrylate adhesive.

iv. Antifibrinolytic rinses, 

k. Scheduled instruction until post-treatment revision.

l. Preventive and maintenance programs of oral hygiene.

-In the dental office, how can be changed the therapeutic approach of antiaggregated and/or anticoagulated patients? (6)

1) Related to the Dental management in patients with classic antiplatelets treatment the material published in the Guidelines of the American College of Chest Physicians about perioperative management of antithrombotic therapy must be followed. Particullary:

a. In patients who are receiving ASA for the secondary prevention of cardiovascular disease and are having minor dental procedures, we suggest continuing ASA around the time of the procedure instead of stopping ASA 7 to 10 days before the procedure.

b. In patients at low risk for cardiovascular events who are receiving ASA therapy, we suggest stopping ASA 7 to 10 days before surgery instead of continuation of ASA.

c. In patients with a coronary stent who are receiving dual antiplatelet therapy and require surgery, we recommend deferring surgery for at least 6 weeks after placement of a bare-metal stent and for at least 6 months after placement of a drug-eluting stent instead of undertaking surgery within these time periods.

d. In patients who require surgery within 6 weeks of placement of a bare-metal stent or within 6 months of placement of a drug-eluting stent, we suggest continuing dual antiplatelet therapy around the time of surgery instead of stopping dual antiplatelet therapy 7 to 10 days before surgery.

2) Related to the Dental management in patients with classic anticoagulants treatment.

a. The studies recommend that in case of simple dental extraction of one or two teeth (contiguous or adjacents), not removing the anticoagulant drug. We must control the drug´s anticoagulant action with the INR, whose value has to be done at least 72 previous hours to the dental extractions, although it´s better if it´s done 24 hours before. The INR ideal value for doing the dental extractions is established between 2 and 4, although it´s widely accepted that the optimal value is in 2,5 because this value minimises the risk of bleeding and thrombosis.

b. If the dental extraction will be complicated, flap and osteotomy is necessary or patients with multiple pathologies we can bridge anticoagulants with low molecular weight heparine, 2 or 3 days before dental surgery. With these patients local hemostatic actions must be used.

3) Related to the dental management in patients with new antiplatelets treatment:

It’s applied the same protocols as the normally used with those patients who take the classic antiplatelet medication. Sometimes may be not effective and therefore there will be a need of a rescue therapy or other therapeutic measures, such as dialysis or gastric lavage.

4) Related to the dental management in patients with new anticoagulants treatment:

a. There are only a few studies about dental management in patients under treatment with the new anticoagulant drugs, although they also indicate the need of making clinic studies that support the protocols.

b. Simple dental extraction and minor surgery have a low risk of bleeding, however the multiple dental extractions have a high risk of bleeding, for this we recommend the dental extraction no more than 3 teeth in the same surgical act and they will be contiguous or adjacents.

c. With the new anticoagulant drugs (dabigatran, rivaroxaban, etc.) it would not be necessary removing its during the dental treatment, although, there are not any studies that support this particular way of acting.

d. New anticoagulants (dabigatran, rivaroxaban and apixaban) have a reduced half life and in case of being necessary its removal, it will be done 24 hours before the surgical treatment. Its reintroduction must be done as soon as possible if there isn’t bleeding, normally recommending to do it 24 hours after surgical act. If there is a postoperative bleeding risk the reintroduction will be done after 2-3 days from surgery with normal dose.

e. At the present, these drugs do not have effective antidote known.

## Consensus report of group d: the hospital dentistry as health resource in special patients

-What kind of patients could benefit from the hospital dental care? (7)

1) Using the ASA scoring system developed by the American Society of Anesthesiologists, In the case of ASA IV patients, dental treatment should be provided in the hospital setting in order to avoid complications. This group includes uncontrolled diabetes; patients with type II angina, patients visiting the dental office with oxygen therapy; individuals with myocardial infarction or stroke in the last 3 months; individuals with a blood pressure of over 200/100 mmHg; patients of very old age, excessive medication use, or the administration of immunosuppressors or anticoagulants.

2) In patients at risk we must ensure good pain control and the use of premedication, sedation or general anesthesia techniques.

-What kind of dental patients have to be treated under general anesthesia in a hospital setting? (8)

1) Patients without underlying systemic diseases (with irrepressible fear, with large needs in a single session).

2) Handicapped Patients (with great difficulty of clinical or behavioral management).

3) Special pediatric patients (patients with nursing bottle caries, extensive rampant caries or severe medical problems).

4) Medically compromised patients (ASA IV patients).

## Consensus report of group e: cardiovascular pathology and dentistry

-Are there any evidence of association between the bacteremia of dental origin and bacterial endocarditis? (9)

1) Dental extraction is the procedure that carries the highest risk of bacteremia in terms of prevalence, duration and magnitude. There is no conclusive evidence that gingival and periodontal health are contributing factors that predispose to the development of bacteremia in patients undergoing dental procedures, although it is likely that they are relevant to the onset of bacteremia when performing periodontal interventions. Activities of everyday living, such as chewing and toothbrushing, can also cause bacteremia and their clinical importance is based on the concept of “cumulative exposure to bacteremia”. As a result of dental treatment a small amount of patients contract bacterial endocarditis.

2) Apart from its possible implication in the onset of episodes of bacterial endocarditis, there has been increasing interest in bacteremia of oral origin in the past two decades due to the major role it is considered to play in the progression of atherosclerosis and consequently in the occurrence of chronic diseases. It is imperative that molecular sequence-based approaches be validated and used in prospective trials to achieve a better understanding of the bacterial characteristics associated with bacteremia of oral origin.

3) Scientific evidence in the field of oral bacteremia has greatly influenced clinical practice guidelines on prophylaxis against bacterial endocarditis of oral origin. The reduction of bacteremia, preventing the adherence of bacteria to the endocardium, is the main benefit of the use of prophylaxis. Over the past 50 years, prophylactic regimens for the prevention of bacterial endocarditis secondary to dental procedures have been modified but remain consensus based. The indication for prophylaxis is now limited to patients with the highest risk of bacterial endocarditis undergoing the highest risk dental procedures. However, the National Institute for Clinical Excellence (NICE) of the United Kingdom has adopted a drastic stance in this respect, recommending the cessation of antibiotic prophylaxis for bacterial endocarditis in individuals undergoing dental procedures in the United Kingdom. Further research should be encouraged to determine the impact of this recommendation of the NICE guideline. In any way, from a legal point of view, it is recommended to follow clinical guidelines established in each country.

4) The conclusions reached by the NICE on the lack of efficacy of antiseptic prophylaxis for the prevention of bacteremia following dental procedures are based on a small volume of published scientific evidence. At the present time, the controversies concerning the efficacy of antibiotic prophylaxis and the risk-benefit and cost-benefit relationships of antibiotic prophylaxis could justify convenience more extensively research on the recommended chlorhexidine regimens and new antiseptic protocols.

5) All Expert Committees on bacterial endocarditis prevention agree on the premise that “Good oral hygiene and regular dental checkups are of particular importance for the prevention of bacterial endocarditis of oral origin”.

-Is there scientific evidence about the relationship between the cardiovascular disease, metabolic syndrome and dental diseases? (10)

1) There is great variability among the studies that have explored the association between oral alterations and cardiovascular disease. Most authors reporting a moderate association, without the firm scientific evidence needed to confirm a true causal relationship.

2) The confirmation of periodontal disease as an independent risk factor would result in more aggressive treatment of patients at a high risk of developing cardiovascular disease. More solid scientific evidence is required in this respect, based on longitudinal studies with standardized measurements and prolonged follow-up, controlling for other risk factors, and randomized studies of periodontal treatment.

3) A recent metaanalysis has revealed a clear association between metabolic syndrome and periodontitis – the subjects with metabolic syndrome being almost twice as likely to develop periodontitis than the rest of the population.

4) The existing scientific evidence suggests that obesity, and particularly diabetes mellitus, could be related to an increased sus-ceptibility to periodontitis. However, it is not clear whether periodontal treatment could improve the systemic conditions of such patients.
